# Concerted suppressive effects of carisbamate, an anti-epileptic alkyl-carbamate drug, on voltage-gated Na^+^ and hyperpolarization-activated cation currents

**DOI:** 10.3389/fncel.2023.1159067

**Published:** 2023-05-24

**Authors:** Te-Yu Hung, Sheng-Nan Wu, Chin-Wei Huang

**Affiliations:** ^1^Department of Pediatrics, Chi Mei Medical Center, Tainan, Taiwan; ^2^Department of Physiology, College of Medicine, National Cheng Kung University, Tainan, Taiwan; ^3^College of Medicine, Institute of Basic Medical Sciences, National Cheng Kung University, Tainan, Taiwan; ^4^School of Medicine, College of Medicine, National Sun Yat-sen University, Kaohsiung City, Taiwan; ^5^Department of Neurology, National Cheng Kung University Hospital, College of Medicine, National Cheng Kung University, Tainan, Taiwan

**Keywords:** carisbamate (RWJ-333369), voltage-gated Na^+^ current, late Na^+^ current, window Na^+^ current, hyperpolarization-activated cation current, current kinetics, antiepileptic drug

## Abstract

Carisbamate (CRS, RWJ-333369) is a new anti-seizure medication. It remains unclear whether and how CRS can perturb the magnitude and/or gating kinetics of membrane ionic currents, despite a few reports demonstrating its ability to suppress voltage-gated Na^+^ currents. In this study, we observed a set of whole-cell current recordings and found that CRS effectively suppressed the voltage-gated Na^+^ (*I*_Na_) and hyperpolarization-activated cation currents (*I*_h_) intrinsically in electrically excitable cells (GH_3_ cells). The effective IC_50_ values of CRS for the differential suppression of transient (*I*_Na(T)_) and late *I*_Na_ (*I*_Na(L)_) were 56.4 and 11.4 μM, respectively. However, CRS strongly decreased the strength (i.e., Δarea) of the nonlinear window component of *I*_Na_ (*I*_Na(W)_), which was activated by a short ascending ramp voltage (*V*_ramp_); the subsequent addition of deltamethrin (DLT, 10 μM) counteracted the ability of CRS (100 μM, continuous exposure) to suppress *I*_Na(W)_. CRS strikingly decreased the decay time constant of *I*_Na(T)_ evoked during pulse train stimulation; however, the addition of telmisartan (10 μM) effectively attenuated the CRS (30 μM, continuous exposure)-mediated decrease in the decay time constant of the current. During continued exposure to deltamethrin (10 μM), known to be a pyrethroid insecticide, the addition of CRS resulted in differential suppression of the amplitudes of *I*_Na(T)_ and *I*_Na(L)_. The amplitude of *I*_h_ activated by a 2-s membrane hyperpolarization was diminished by CRS in a concentration-dependent manner, with an IC_50_ value of 38 μM. For *I*_h_, CRS altered the steady-state *I–V* relationship and attenuated the strength of voltage-dependent hysteresis (Hys_(V)_) activated by an inverted isosceles-triangular *V*_ramp_. Moreover, the addition of oxaliplatin effectively reversed the CRS-mediated suppression of Hys_(V)_. The predicted docking interaction between CRS and with a model of the hyperpolarization-activated cyclic nucleotide-gated (HCN) channel or between CRS and the hNa_V_1.7 channel reflects the ability of CRS to bind to amino acid residues in HCN or hNa_V_1.7 channel via hydrogen bonds and hydrophobic interactions. These findings reveal the propensity of CRS to modify *I*_Na(T)_ and *I*_Na(L)_ differentially and to effectively suppress the magnitude of *I*_h_. *I*_Na_ and *I*_h_ are thus potential targets of the actions of CRS in terms of modulating cellular excitability.

## 1. Introduction

Carisbamate (CRS, RWJ-333369), a bioactive, orally administered neuromodulator, has been demonstrated to be beneficial for the treatment of different types of convulsive disorders, including drug-resistant focal epilepsy and partial-onset seizure (Whalley et al., 2009; Sperling et al., 2010; Vohora et al., 2010; Ono et al., 2011; Faure et al., 2013, 2014; Fernandes et al., 2015; Marques-Carneiro et al., 2017; Steriade et al., 2020; Löscher et al., 2021; Lu et al., 2021; Strzelczyk and Schubert-Bast, 2021; Elkommos and Mula, 2022; Pong et al., 2022). This compound was also reported to be effective in the treatment of alcoholism (Rezvani et al., 2009, 2012). One study found that CRS prevented the development and production of epilepsy-like discharges and exerted a neuroprotective effect after epilepticus-like injury (Fernandes et al., 2015).

CRS was demonstrated to suppress glutamate transmission in granule cells of the dentate gyrus (Lee et al., 2011). It also was reported to effectively suppress action potential firing and exert neuroprotective effects in either cultured hippocampal neurons or a rat model of status epilepticus (Deshpande et al., 2008a,b, 2016a,b; Fernandes et al., 2015; Deshpande and DeLorenzo, 2020). Alternatively, CRS can inhibit voltage-gated Na^+^ channels (Liu Y. et al., 2009; Whalley et al., 2009; Vohora et al., 2010; Shim et al., 2013). One paper reported the ability of CRS to suppress T-type voltage-gated Ca^2+^ currents (Kim et al., 2017). Hence, it remains unclear whether and how CRS can perturb ionic currents.

The magnitude of the voltage-gated Na^+^ current (*I*_Na_) through voltage-gated Na^+^ (Na_V_) channels is crucial to the generation and propagation of action potentials in excitable membranes (Catterall et al., 2020; Menezes et al., 2020). During abrupt depolarization, these channels become activated and can shift quickly from a resting (or closed) to an open state, thereby allowing the flow of Na^+^ ions from the extracellular solution to the cell interior under electrochemical driving forces. Na^+^ channels that have been opened in a voltage-dependent manner then shift to an inactive state, resulting in the transient enhancement of *I*_Na_. In addition to the voltage dependence of slow *I*_Na_ inactivation, studies have reported the striking cumulative inhibition of the current activated during pulse train stimulation (Taddese and Bean, 2002; Huang et al., 2015; Navarro et al., 2020; Lin et al., 2022; Wu et al., 2022a). At present, it remains unclear whether and how CRS can interact with Na_V_ channels to modify the magnitude, gating kinetics, and frequency-dependence of *I*_Na_.

The hyperpolarization-activated cation current, *I*_h_ (i.e., the funny current [*I*_f_]), is a key determinant of repetitive electrical activity in heart cells and various types of central neurons and endocrine or neuroendocrine cells (Belardinelli et al., 1988; Irisawa et al., 1993; Simasko and Sankaranarayanan, 1997; Stojilkovic et al., 2010; Kretschmannova et al., 2012; Wu and Huang, 2021; Choi et al., 2022; Chuang et al., 2022; Depuydt et al., 2022; Peters et al., 2022). This ionic current has unusually slow voltage-dependent activation kinetics and is a mixed, inwardly directed Na^+^/K^+^ current that is sensitive to blocking by CsCl or ivabradine (Cao et al., 2016; Hsiao et al., 2019). Activation of *I*_h_ may cause the resting potential to depolarize and, in turn, reach the threshold required to generate or elicit an action potential; as a result, *I*_h_ can affect pacemaker activity and impulse propagation (Anastasaki et al., 2022; Lei et al., 2022; Peters et al., 2022). Moreover, the slow kinetics of *I*_h_ in response to a long hyperpolarizing step can produce long-lasting, activity-dependent modification of membrane excitability in different excitable cell types (Kretschmannova et al., 2012; Peters et al., 2021; Mäki-Marttunen and Mäki-Marttunen, 2022). *I*_h_ is transmitted through channels encoded by members of the hyperpolarization-activated cyclic nucleotide-gated (*HCN*) gene family, and studies have shown that the activity of these channels is responsible for the ionic mechanisms of different convulsive disorders (Noam et al., 2011; Banarroch, 2013; Concepcion et al., 2021).

In this study, we extensively investigated the electrophysiological actions of CRS in excitable cells with consideration of the above-described information. We aimed to investigate the intrinsic effects of CRS on the magnitude and/or gating of *I*_Na_ or *I*_h_ in GH_3_ neuronal cells. Our findings prompt us to consider both *I*_Na_ and *I*_h_ in excitable cells as distinctive targets of CRS, through which it can act concertedly to influence the functional activities of the cells involved.

## 2. Materials and methods

### 2.1. Chemicals, drugs, reagents, and solutions used in this work

Carisbamate (CRS; also known as Comfyde™, RWJ-333369, YKP 509, [(2*S*)-2-(2-chlorophenyl)-2-hydroxyethyl] carbamate, (*S*)-2-*O*-carbamoyl-1-*o*-chlorophenyl-ethanol, C_9_H_10_ClNO_3_) and deltamethrin (DLT) were acquired from MedChemExpress (MCE^®^; Everything Biotech, New Taipei City, Taiwan). Telmisartan (Tel) was obtained from Tocris Cookson Ltd. (Bristol, UK). Oxaliplatin (OXAL) was obtained from Sanofi-Aventis (New York, NY, USA). Nimodipine, tetraethylammonium chloride (TEA), and tetrodotoxin (TTX) were obtained from Sigma-Aldrich (St. Louis, MO, USA). Chlorotoxin was a gift from Prof. Dr. Woei-Jer Chuang (Department of Biochemistry, National Cheng Kung University Medical College, Tainan, Taiwan). Other chemicals (e.g., CsCl, CsOH, and CdCl_2_) and reagents used in this work were obtained from commercial sources and of reagent grade. Reagent-grade water was de-ionized using a Milli-Q^®^ water purification system (Millipore, Bedford, MA, USA).

HEPES-buffered normal Tyrode’s solution was used as the electrophysiological bath solution, with an ionic composition of (in mM): NaCl, 136.5; KCl, 5.4; MgCl_2_, 0.53; CaCl_2_, 1.8; glucose, 5.5; and HEPES-NaOH buffer, 5.5 (pH 7.4). The intracellular pipette solution used to measure the ions flowing through the whole-cell K^+^ current or hyperpolarization-activated inward current (*I*_h_) had the following composition (in mM): Potassium aspartate, 130; KCl, 20; MgCl_2_, 1; Na_2_ATP, 3; Na_2_GTP, 0.1; EGTA, 0.1; and HEPES-KOH buffer, 5 (pH 7.2). To record different types of voltage-gated Na^+^ current (*I*_Na_), we replaced K^+^ ions in the internal solution with equimolar Cs^+^ ions and titrated the pH value to 7.2 by adding CsOH; the studied cells were commonly bathed in Ca^2+^-free Tyrode’s solution containing 10 mM TEA. Generally, the bath and pipette solutions were filtered on the day of use through a syringe filter equipped with a 0.22-μm Supor^®^ nylon membrane (#4612; Pall Corp.; Genechain, Kaohsiung, Taiwan).

### 2.2. Cell preparation

The GH_3_ pituitary cell line (BCRC-60015) was obtained from the Bioresources Collection and Research Center (Hsinchu, Taiwan). The cells were maintained in 50-mL plastic culture flasks in Ham’s F-12 medium supplemented with 2.5% fetal calf serum (*v/v*), 15% horse serum (*v/v*), and 2 mM glutamine (Wu et al., 2017; Hung et al., 2021). The medium was replaced twice weekly, and the cells were carefully split into subcultures once weekly. The cells were subjected to electrophysiological measurements when they reached 60%–80% confluence (5 or 6 days after subculture).

### 2.3. Electrophysiological measurements (patch-clamp recordings)

Shortly before each measurement, the GH_3_ cells were carefully dispersed. A few drops of the cell suspension were rapidly transferred to a custom-built chamber, and the cells were allowed to settle on the bottom surface. The chamber used to record was tightly mounted on the stage of an inverted phase-contrast microscope (Diaphot-200; Nikon; Lin Trading Co., Taipei, Taiwan) coupled to a video camera system (magnification: 1500×) to monitor cell size during the experiments. The cells were kept at room temperature (20–25°C) in a bath of normal Tyrode’s solution containing 1.8 mM CaCl_2_. The patch electrodes were carefully drawn from Kimax-51 capillaries with an outer diameter of 1.5–1.8 mm (#34500; Kimble; Dogger, New Taipei City, Taiwan) using a two-stage PP-83 puller (Narishige; Taiwan Instrument, Tainan, Taiwan), and the electrode tips were fire-polished with an MF-83 microforge (Narishige). Once filled with different pipette solutions as described in the section “2.1. Chemicals, drugs, reagents, and solutions used in this work,” the tip resistance of the electrodes generally ranged from 3 to 5 MΩ. We used an RK-400 patch amplifier (Bio-Logic, Claix, France) to perform standard patch-clamp recordings in a modified whole-cell configuration (Lai et al., 2020, 2022). Junction potentials, which develop at the electrode tip when the composition of the internal solution differs from that in the bath, were nullified shortly before seal formation was made, and junction potential corrections were applied to the whole-cell data. While recording, the signal output data (i.e., potential or current tracings) were stored online at a frequency of ≥10 kHz using an ASUSPRO-BN401 LG laptop computer (ASUS; Yun-Dai, Tainan, Taiwan) equipped with a Digidata 1440A device (Molecular Devices; Advanced Biotech, New Taipei City, Taiwan) and controlled by pCLAMP 10.6 software (Molecular Devices).

### 2.4. Data analyses

We constructed and then analyzed the concentration–response curves of CRS-mediated inhibition on the peak (transient, *I*_Na(T)_) and sustained (late, *I*_Na(L)_) components of depolarization-activated *I*_Na_ and the hyperpolarization-activated inward current (*I*_h_) present in GH_3_ cells. The *I*_Na_ was evoked by a 30-ms depolarizing pulse to −10 mV from a holding potential of −100 mV, and the current amplitudes were measured at the start (*I*_Na(T)_) and end (*I*_Na(L)_) of the depolarizing pulse in the presence or absence of exposure to different CRS concentrations. The *I*_h_ was activated slowly by a 2-s hyperpolarizing step from −40 to −110 mV, and the current amplitude was measured at the end of the hyperpolarizing command voltage. The concentration of CRS needed to inhibit 50% of the current amplitude (IC_50_) was approximated using the three-parameter logistic model, a modified form of the sigmoidal Hill equation, with goodness-of-fitness assessments as follows:


R⁢e⁢l⁢a⁢t⁢i⁢v⁢e⁢a⁢m⁢p⁢l⁢i⁢t⁢u⁢d⁢e=

$$\,\left\{{\left[C\,R\,S\right]}^{-n_{H}}\times \left(1-a\right)\right\}/\left\{{\left[C\,R\,S\right]}^{-n_{H}}+I\,C_{50}^{-n_{H}}\right\}+a$$

In this equation, [*CRS*] = the CRS concentration, *n*_*H*_ = the Hill coefficient, and 1 *– a* represents the maximal inhibition.

### 2.5. Curve-fitting procedures and statistical analyses

Linear or nonlinear (e.g., exponential of sigmoidal function) regression was used to fit continuous curves to the experimental data, using pCLAMP 7 software (Molecular Devices), 64-bit OriginPro 2022b software (OriginLab^®^; Scientific Formosa, Kaohsiung, Taiwan), or Excel 2016 software (Microsoft, Redmond, WA, USA) with the “Solver” add-in function. The experimental data consist of different types of ionic currents and are presented as the mean ± standard error of the mean (SEM). The sample size (n) indicates the number of cells from which data were collected; and SEM error bars were plotted. The Kolmogorov Smirnov test for normality indicated that the data distribution obtained was satisfactory. To assess differences between more than two groups, we used an analysis of variance (ANOVA-1 or ANOVA-2) with or without repeated measures, followed by a *post-hoc* Fisher’s least significant difference test. Differences were considered statistically significant at a *p*-value < 0.05.

### 2.6. Docking studies

To explore the docking prediction, we first selected the structure from PDB (PDB: 5V4S) and entered it into the PyRx software. We also selected the chemical structure from PubChem (Compound CID: 6918474) and entered it into PyRx. After that, we performed appropriate docking actions in PyRx. Subsequently, we selected the results of this interaction and used LigPLOT+ software to generate an image that predicted the docking.

## 3. Results

### 3.1. Suppressive effect of carisbamate on the voltage-gated Na^+^ current (*I*_Na_)

**FIGURE 1:**
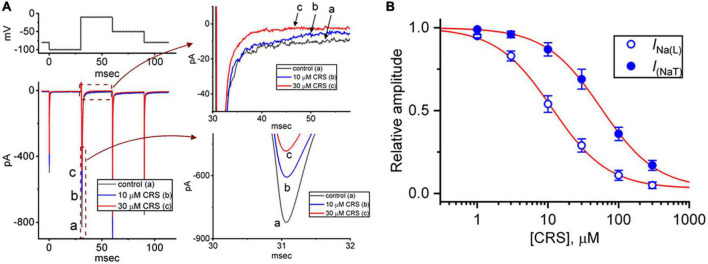
Inhibitory effects of carisbamate (CRS) on the transient (peak, *I*_Na_**_(T)_**) or late (sustained, *I*_Na_**_(L)_**) component of the voltage-gated Na^+^ current (*I*_Na_). **(A)** The exemplar current traces shown at left were acquired in the control period without CRS (a, black line) and during exposure to 10 μM (b, blue line) or 30 μM CRS (c, red line). The voltage-clamp protocol is illustrated at the top left, while the current traces shown at right represent expansions of the records from the dashed boxes in the left side of panel **(A)**. **(B)** Concentration-dependent CRS-induced inhibition of *I*_Na(T)_ (filled blue circles) and *I*_Na(L)_ (open blue circles) in GH_3_ cells. Each data point indicates the mean ± standard error of the mean (*n* = 8). The red sigmoidal lines, on which the data points are overlaid, indicate the best fit to a modified Hill equation according to the averaged data obtained at each concentration of CRS, as elaborated in the section “2. Materials and methods”. The IC_50_ values required for CRS-mediated inhibition of *I*_Na(T)_ and *I*_Na(L)_ were 56.4 and 11.4 μM, respectively.

In our initial whole-cell experiments, we examined whether CRS exerted any effects on *I*_Na_ in pituitary GH_3_ cells. To avoid contamination by Ca^2+^ currents, the cells were bathed in Ca^2+^-free Tyrode’s solution containing 10 mM TEA and 0.5 mM CdCl_2_, and the recording electrode was filled with a Cs^+^-containing internal solution. In the recording pipette, Cl^–^ ions were replaced with aspartate to eliminate any possible contamination by Cl^–^ currents, because CRS was reported to alter the magnitude of Cl^–^ currents (Whalley et al., 2009). As demonstrated in Figure 1A, after exposure of GH_3_ cells to CRS for 1 min at a concentration of 10 or 30 μM, the amplitudes of *I*_Na(T)_ and *I*_Na(L)_ decreased from the control values of 813 ± 15 pA (*n* = 7) and 9.7 ± 0.3 pA (*n* = 7), respectively, to 598 ± 11 pA (*n* = 7, *p* < 0.05) and 4.4 ± 0.2 pA (*n* = 7, *p* < 0.05), and to 485 ± 9 pA (*n* = 7, *p* < 0.05) and 2.5 ± 0.1 pA (*n* = 7, *p* < 0.05), respectively. After the removal of CRS, the amplitudes of *I*_Na(T)_ and *I*_Na(L)_ returned to 801 ± 14 (*n* = 7, *p* < 0.05) and 9.5 ± 0.2 pA (*n* = 7, *p* < 0.05), respectively. Continuous exposure of the cells to 1 μM TTX alone, but not to 1 μM nimodipine alone, nearly eliminated the *I*_Na(T)_ amplitude in response to the same voltage protocol, as demonstrated by a reduction from the control value of 814 ± 16 pA to 31 ± 5 pA (*n* = 7, *p* < 0.01). Concomitant with changes in the amplitude of *I*_Na(T)_ or *I*_Na(L)_, CRS (30 μM) decreased the time constant for the slow component of *I*_Na(T)_ inactivation (τ_inact(S)_) from 9.5 ± 0.4 to 4.2 ± 0.4 ms (*n* = 7, *p* < 0.05); however, no clear change in the time constant for the fast component of current inactivation was observed.

Figure 1B demonstrates that exposure to CRS can decrease the amplitudes of *I*_Na(T)_ and *I*_Na(L)_ evoked by a rapid depolarizing pulse in a concentration-dependent manner. According to the modified Hill equation detailed in the section “2.4. Data analyses,” the IC_50_ value needed for CRS-mediated suppression of *I*_Na(T)_ or *I*_Na(L)_ from GH_3_ cells was calculated as 56.4 or 11.4 μM, respectively, and these distinct values demonstrate the difference between the inhibitory effects of CRS on the magnitudes of *I*_Na(T)_ and *I*_Na(L)_ in these cells. Therefore, as observed during a rectangular depolarizing step, this drug exhibits a preferential inhibition of *I*_Na(L)_ over *I*_Na(T)_ when activated.

### 3.2. Effect of CRS on the steady-state current versus voltage (*I–V*) relationship of *I*_Na(T)_

**FIGURE 2:**
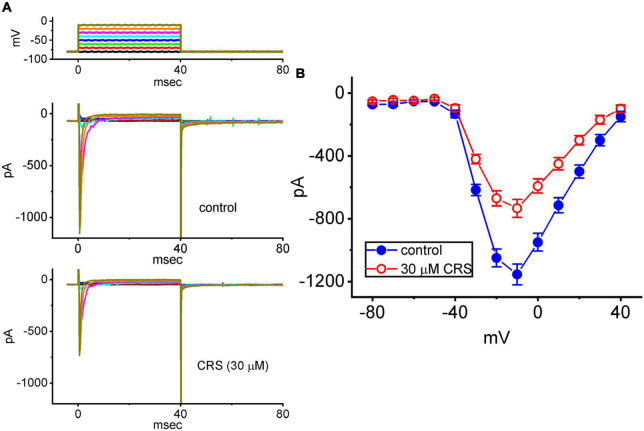
Inhibitory effect of CRS on the steady-state current versus voltage (*I–V*) relationship of the transient peak component of the voltage-gated Na^+^ current (*I*_Na_**_(T)_**). **(A)** Exemplar current traces acquired in pituitary GH_3_ cells in the control period without CRS (upper) and during exposure to 30 μM CRS (lower). The voltage-clamp protocol is illustrated at the top, and the potential traces shown in different colors correspond to the current traces evoked by the same levels of step commands. **(B)** Averaged *I–V* relationships of *I*_Na(T)_ acquired in the absence (blue filled circles) and presence (red open circles) of 30 μM CRS. Each point represents the mean ± standard error of the mean (*n* = 8). The current amplitude was obtained at the beginning of each depolarization step from a holding potential of –80 mV.

To further characterize the inhibitory effect of CRS, we explored whether this drug could alter the steady-state *I–V* relationship of *I*_Na(T)_ in GH_3_ cells. Figure 2 illustrates the average *I–V* relationship of *I*_Na(T)_ in the presence or absence of 30 μM CRS. Notably, the value of the threshold or maximal voltage required to elicit *I*_Na(T)_ did not differ between the absence and presence of CRS. Accordingly, we conclude that continuous exposure to CRS does not elicit an obvious change in the quasi-steady-state *I–V* relationship of *I*_Na(T)_, despite a significant reduction in *I*_Na(T)_ conductance.

### 3.3. Suppressive effect of CRS on the window component of *I*_Na_ (*I*_Na(W)_)

**FIGURE 3:**
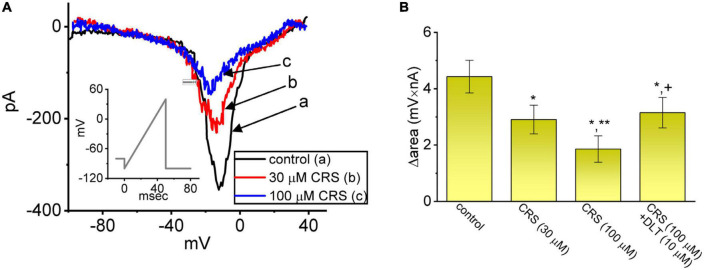
Modification by carisbamate (CRS) of a nonlinear window *I*_Na_ (*I*_Na_**_(W)_**) in response to a short ascending ramp voltage (*V*_ramp_). **(A)** Exemplar current traces (i.e., the relationship of current amplitude versus non-linear membrane potential) obtained in the control period without CRS (a, black) and during exposure to 30 μM (b, red) or 100 μM CRS (c, blue). The voltage protocol is illustrated in the inset graph; the downward deflection denotes the instantaneous inward current (i.e., *I*_Na(W)_) activated by a short ascending *V*_ramp_. **(B)** Bar graph summarizing the effect of CRS (30 or 100 μM) and CRS (100 μM) plus deltamethrin (DLT, 10 μM) on the Δarea of *I*_Na(W)_ (mean ± standard error of the mean; *n* = 7 for each bar). The Δarea was measured at voltages between –80 and +40 mV during the ascending *V*_ramp_. *Significantly different from the control group (*p* < 0.05), **significantly different from the CRS (30 μM) alone group (*p* < 0.05), and ^+^significantly different from the CRS (100 μM) alone group (*p* < 0.05).

Instantaneous *I*_Na(W)_, which is activated in response to an upsloping (or ascending) ramp voltage (*V*_ramp_), has been demonstrated in different types of excitable cells (Morris et al., 2012; Yu et al., 2012; Wu et al., 2022a). We next examined whether CRS could alter the magnitude of *I*_Na(W)_ evoked by a rapidly ascending *V*_ramp_. In this set of experiments, we voltage-clamped the studied cell at −80 mV and applied an ascending *V*_ramp_ from −100 to +40 mV for a duration of 50 ms (ramp speed = 2.8 mV/ms) to activate *I*_Na(W)_. Within 1 min of exposing the cells to CRS (30 or 100 μM), the magnitude (i.e., Δarea) was strikingly decreased due to the 50-ms ascending *V*_ramp_ (Figure 3). For example, exposure to 30 or 100 μM CRS led to considerable attenuation of the Δarea of *I*_Na(W)_ from a control value of 4.43 ± 0.58 mV⋅nA (*n* = 7) to 2.91 ± 0.51 mV⋅nA (*n* = 7, *p* < 0.05) or 1.86 ± 0.47 mV⋅nA (*n* = 7, *p* < 0.05), respectively. Deltamethrin (DLT), a type II pyrethroid, was reported to activate *I*_Na_ (Bothe and Lampert, 2021). Under continuous exposure to 100 μM CRS, the exposure of cells to 10 μM DLT effectively increased the Δarea to 3.15 ± 0.54 mV⋅nA (*n* = 7, *p* < 0.05), indicating that CRS-mediated increase in *I*_Na(W)_ can be partially restored by further addition of DLT. Our results also confirm that CRS primarily inhibits *I*_Na(W)_ itself, rather than other types of voltage-gated ionic currents.

### 3.4. CRS-mediated increase in the cumulative inhibition of *I*_Na(T)_ inactivation

**FIGURE 4:**
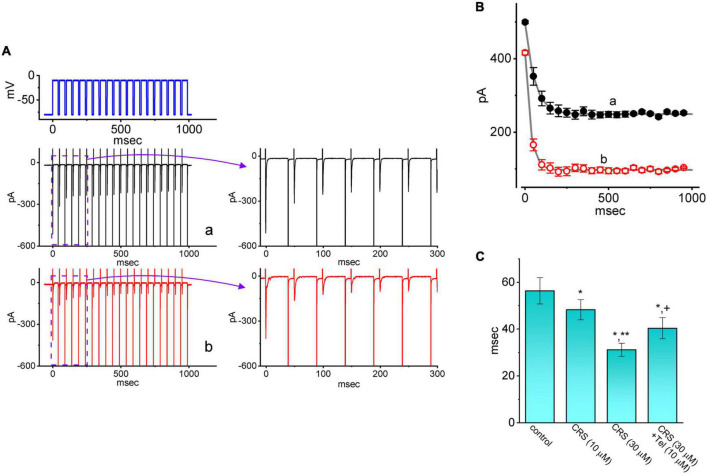
Effect of carisbamate (CRS) on the transient peak component of the voltage-gated Na^+^ current (*I*_Na_**_(T)_**) during a train of depolarizing command voltages. **(A)** Exemplar current traces during the control period without CRS (a, black color) or exposure to 30 μM CRS (b, red color) are shown at left. The upper left graph depicts the applied voltage-clamp protocol. The graph at right illustrates the expanded record from the purple dashed box at left. **(B)** The relationship between the *I*_Na(T)_ amplitude and pulse duration without CRS (a, black filled circles) and during exposure to 30 μM CRS (b, red open circles) (mean ± standard error of the mean [SEM]; *n* = 7 for each point). Each smooth gray line is well fitted to a single exponential. **(C)** Bar graph summarizing the effect of CRS (10 or 30 μM) and CRS (30 μM) plus telmisartan (Tel, 10 μM) on the decaying time constant (τ) of *I*_Na(T)_ elicited by repetitive depolarizing command voltages (mean ± SEM; *n* = 7 for each bar). *Significantly different from the control group (*p* < 0.05), **significantly different from the CRS (10 μM) alone group (*p* < 0.05), and ^+^significantly different from the CRS (30 μM) alone group (*p* < 0.05).

*I*_Na(T)_ inactivation was recently shown to accumulate during repetitive short pulses, and this accumulation is thought to be responsible for various electrical behaviors (Huang et al., 2015; Navarro et al., 2020). Therefore, an additional series of measurements was performed to ascertain whether CRS could perturb *I*_Na(T)_ inactivation following activation by a train of depolarizing stimuli. The studied cell was held at a voltage of −80 mV and subjected to the following stimulus protocol: repetitive depolarization from −10 mV (40 ms per pulse at a rate of 20 Hz for 1 s). *I*_Na(T)_ inactivation in GH_3_ cells was evoked by 1-s repetitive depolarization from −80 to −10 mV with a decay time constant of 56.4 ± 5.6 ms (*n* = 7), which was obtained during the control period (i.e., absence of CRS) (Figure 4). In other words, a rapid current decay occurred as a function of time in a single-exponential manner. In particular, while exposing cells to 10 or 30 μM CRS, the decay time constant of *I*_Na(T)_ activated by the same train of depolarizing stimuli decreased considerably to 48.3 ± 4.3 ms (*n* = 7, *p* < 0.05) or 31.2 ± 2.8 ms (*n* = 7, *p* < 0.05), respectively. Tel, an antagonist of the angiotensin II receptor, has been recognized as an effective activator of *I*_Na_ (Chang et al., 2018; Chang and Wu, 2018; Lai et al., 2020). The addition of Tel (10 μM) increased the decay time constant of the current to 40.4 ± 4.5 ms (*n* = 7, *p* < 0.05). Our results confirm that that CRS primarily inhibits Na^+^ current, which show cumulative inhibition, rather than other type of voltage-gated ionic currents.

### 3.5. Attenuating effect of CRS on the DLT-mediated increase in *I*_Na_

**FIGURE 5:**
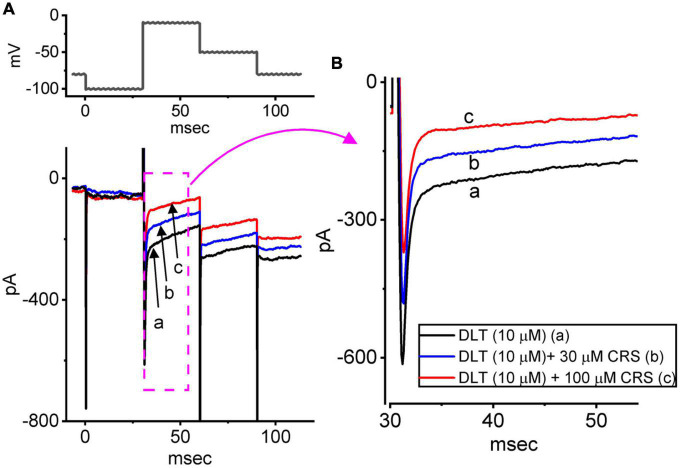
Effects of deltamethrin (DLT) alone and plus carisbamate (CRS) on the voltage-gated Na^+^ current (*I*_Na_). The cells were bathed in Ca^2+^-free Tyrode’s solution containing 10 mM tetraethylammonium chloride and 0.5 mM CdCl_2_, and the measuring electrode was filled with a Cs^+^-enriched solution. **(A)** Exemplar current traces obtained in the presence of DLT (10 μM) alone (a, black), DLT (10 μM) plus CRS (30 μM), or DLT (10 μM) plus CRS (100 μM). **(B)** Expanded records from the pink dashed box in panel **(A)**. Of note, exposure to CRS effectively attenuated DLT-mediated stimulation of *I*_Na_**_(T)_** and *I*_Na_**_(L)_** in GH_3_ cells.

CRS was shown to effectively modify the magnitude of *I*_Na_ (Whalley et al., 2009), while DLT was previously reported to be a pyrethroid insecticide that can activate *I*_Na_ (Bothe and Lampert, 2021; Lin et al., 2022). Therefore, we next investigated whether the presence of CRS could modify the DLT-enhanced *I*_Na_ evoked in GH_3_ cells by a rapid depolarizing pulse. In this set of experiments, when establishing whole-cell current recordings, we voltage-clamped the examined cell at −100 mV and applied a 30-ms depolarizing pulse to −10 mV. In keeping with previous observations (Liu Y. et al., 2009; Whalley et al., 2009; Vohora et al., 2010), we found that exposing cells to 10 μM DLT alone for 1 min enhanced the amplitude of *I*_Na(T)_ or *I*_Na(L)_ (Figure 5). However, the subsequent addition of CRS attenuated this effect of DLT on the depolarization-mediated activation of *I*_Na(T)_ or *I*_Na(L)_. For example, as the cells were depolarized from −100 to −10 mV to evoke *I*_Na_, exposing the cells to 10 μM DLT resulted in a considerable increase in *I*_Na(T)_ or *I*_Na(L)_ from a control value of 412 ± 14 pA (*n* = 8) or 89 ± 9 pA (*n* = 8), respectively, to 607 ± 18 pA (*n* = 8, *p* < 0.05) or 178 ± 13 pA (*n* = 8, *p* < 0.05), respectively. Moreover, the further addition of 10 μM CRS to the cells, which remained under continuous exposure to DLT, greatly reduced *I*_Na(T)_ or *I*_Na(L)_ to 478 ± 15 pA (*n* = 8, *p* < 0.05) or 141 ± 11 pA (*n* = 8, *p* < 0.05), respectively. In this regard, the experimental data reflect that CRS could modify the DLT-mediated enhancement of the magnitude of *I*_Na(T)_ and *I*_Na(L)_ in GH_3_ cells.

### 3.6. Effect of CRS on the amplitude of the hyperpolarization-activated cation current (*I*_h_) in GH_3_ cells

**FIGURE 6:**
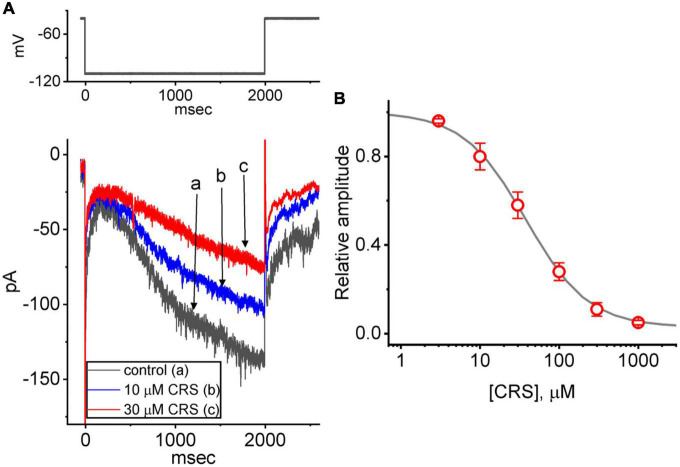
Effect of carisbamate (CRS) on the hyperpolarization-activated cation current (*I*_h_). This set of measurements was obtained from cells bathed in Ca^2+^-free Tyrode’s solution containing 1 μM tetrodotoxin, and the measuring electrode was filled with a K^+^-enriched solution. **(A)** Exemplar current traces (i.e., *I*_h_) acquired in the control period without CRS (a, black line) and during exposure to 10 μM (b, blue line) or 30 μM CRS (c, red line). The upper graph depicts the applied voltage-clamp protocol. **(B)** Concentration-dependent CRS-mediated suppression of *I*_h_ activation via 2-s long-lasting membrane hyperpolarization (mean ± standard error of the mean, *n* = 8 for each point). The gray sigmoidal line, on which the data points are overlaid, indicates the best fit to a modified Hill equation as elaborated in the Materials and methods. The current amplitude was obtained at different CRS concentrations (3 μM–1 mM) at the end of a 2-s period of hyperpolarizing command voltage to –110 mV from a holding potential of –40 mV. The IC_50_ value required for CRS-mediated inhibition of *I*_h_ was 38 μM.

We further investigated the effects of CRS and other relevant compounds on *I*_h_. In these experiments, we placed cells in Ca^2+^-free Tyrode’s solution and filled the recording electrode with a K^+^-enriched solution. We then applied long-lasting step hyperpolarization to activate an inwardly directed current with slowly activating and deactivating properties. Studies have identified this type of ionic current as the *I*_h_ or *I*_f_ (Figure 6A) (Belardinelli et al., 1988; Irisawa et al., 1993; Simasko and Sankaranarayanan, 1997; Liu Y. C. et al., 2009; Wu et al., 2022b; Chuang et al., 2022). Within 1 min of exposing the GH_3_ cells to CRS (10 or 30 μM), the *I*_h_ amplitude in response to such membrane hyperpolarization exhibited an evident decrease. For example, as the cells were hyperpolarized from −40 to −110 mV for a duration of 2 s, exposure to CRS at 10 or 30 μM noticeably reduced the amplitude of the late inward current at the end of hyperpolarization from a control value of 136 ± 18 pA (*n* = 8) to 103 ± 17 pA (*n* = 8, *p* < 0.05) or 73 ± 12 pA (*n* = 8, *p* < 0.05), respectively. Upon returning to −40 mV, the amplitude of the deactivating *I*_h_ decreased from a control value of 93 ± 13 pA (*n* = 8) to 73 ± 12 pA (*n* = 8, *p* < 0.05) or 51 ± 9 pA (*n* = 8, *p* < 0.05) under exposure to 10 or 30 μM CRS, respectively. As shown in Figure 6A, the activation time constant (τ_act_) of *I*_h_ elicited during long membrane hyperpolarization also increased, as evidenced by a significant rise in the τ_act_ value from 212 ± 12 to 402 ± 19 m (*n* = 8, *p* < 0.05) in the presence of 30 μM CRS. This suppression of *I*_h_ in GH_3_ cells was readily reversed upon the removal of CRS.

The relationship between the concentration of CRS and the relative amplitude of *I*_h_ is illustrated in Figure 6B. Exposure to CRS was found to reduce the amplitude of *I*_h_ during long membrane hyperpolarization in a concentration-dependent manner. Using the modified Hill equation, the IC_50_ value required for CRS to inhibit the *I*_h_ amplitude as seen in GH_3_ cells was estimated to be 38 μM; 1 mM CRS almost completely inhibited the current amplitude.

**FIGURE 7:**
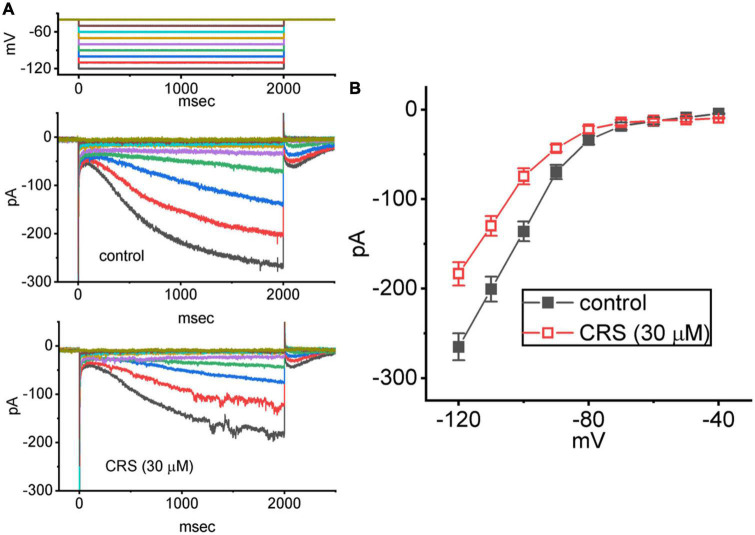
Inhibitory effect of carisbamate (CRS) on the average steady-state current versus voltage (*I–V*) relationship of the hyperpolarization-activated cation current (*I*_h_). The cells were bathed in Ca^2+^-free Tyrode’s solution, and a series of 2-s voltage steps between –120 and –40 mV (10 mV per step) was delivered to each studied cell. **(A)** Exemplar current traces obtained in the control period without CRS (upper) and during exposure to 30 μM CRS. The upper graph depicts the voltage-clamp protocol applied to the tested cell. Potential traces shown in different colors correspond to the ionic currents that were evoked by the same level of command voltage. **(B)** Average *I–V* relationship of *I*_h_ acquired in the absence (filled black squares) and presence (open red squares) of 30 μM CRS (mean ± standard error of the mean; *n* = 8 for each point). The current amplitude was measured at the end of each voltage step with a duration of 2 s.

We further studied the average *I–V* relationship for *I*_h_ in the presence or absence of 30 μM CRS. As shown in Figures 7A, B, this *I–V* relationship exhibited robust, inwardly rectifying non-ohmic behavior. Under exposure to CRS, the *I*_h_ amplitude in the cells was depressed throughout the voltage clamp step. For example, CRS exposure markedly decreased the whole-cell *I*_h_ conductance, measured between −120 and −100 mV, from 6.9 ± 0.2 to 5.3 ± 0.2 nS (*n* = 8, *p* < 0.05); after washout of CRS, the *I*_h_ conductance was restored to 6.7 ± 0.2 nS (*n* = 7, *p* < 0.05).

### 3.7. Suppressive effect of CRS on the voltage-dependent hysteresis (Hys_(V)_) of *I*_h_ evoked in response to a long-lasting triangular *V*_ramp_

**FIGURE 8:**
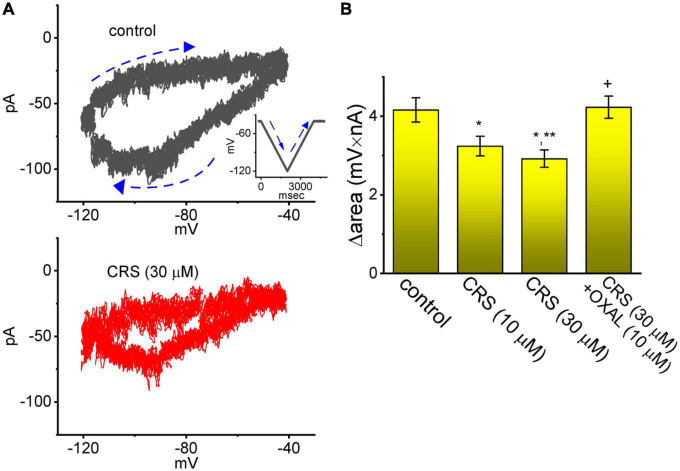
Attenuating effect of carisbamate (CRS) on the voltage-dependent hysteresis (Hys**_(V)_**) of the hyperpolarization-activated cation current (*I*_h_). **(A)** Exemplar Hys_(V)_ (i.e., current trace versus ramp voltage [V_ramp_]) acquired in the absence (upper graph, gray line) and presence (lower graph, red line) of 30 μM CRS. The inset in the upper graph depicts the applied *V*_ramp_ protocol. Dashed arrows show the direction of *I*_h_ or the voltage trace in which time passes during the elicitation of an inverted triangular V_ramp_. **(B)** Bar graph summarizing the effects of CRS (10 or 30 μM) and CRS (30 μM) plus oxaliplatin (OXAL, 10 μM) on the Δarea of the Hys_(V)_ of *I*_h_ (mean ± standard error of the mean; *n* = 8 for each yellow bar). The Δarea, indicated as a function of the strength of the window component of the voltage-gated Na^+^ current (*I*_Na_**_(W)_**), was calculated under the encircled area, which was activated during the downsloping (descending) and upsloping (ascending) ends of the triangular *V*_ramp_. Note the *V*_ramp_-induced Hys_(V)_ for *I*_h_ elicitation; the strength of this Hys_(V)_ was attenuated by adding CRS. *Significantly different from the control group (*p* < 0.05), **significantly different from the CRS (10 μM) alone group (*p* < 0.05), and ^+^significantly different from the CRS (30 μM) alone group (*p* < 0.05).

The Hys_(V)_ behavior of *I*_h_ has been shown to strongly influence electrical behaviors (i.e., action potential firing) in different types of excitable cells (Mãnnikkõ et al., 2005; Fürst and D’Alvanzo, 2015; Chan et al., 2020; Chuang et al., 2022; Wu et al., 2022b). For this reason, we explored whether CRS could perturb the Hys_(V)_ behavior of *I*_h_. In this stage of whole-cell experiments, we applied a long-lasting inverted triangular *V*_ramp_ for a duration of 2 s (i.e., with a ramp pulse of ±40 mV/s) to measure the Hys_(V)_ properties. As shown in Figure 8, the trajectory of *I*_h_ activation in response to the downsloping (i.e., hyperpolarization from −40 to −120 mV) and upsloping (depolarization from −120 to −40 mV) limbs of *V*_ramp_ as a function of time was distinguishable. The current magnitude in response to the downsloping (descending) limb of the inverted triangular *V*_ramp_ was much larger than that evoked by the upsloping (ascending) limb. In terms of the relationship between *I*_h_ amplitude and membrane potential, we clearly observed the Hys_(V)_ behavior of the current amplitude in a clockwise direction. Interestingly, when the GH_3_ cells were continually exposed to CRS, the Hys_(V)_ strength of *I*_h_ evoked by the inverted triangular *V*_ramp_ progressively decreased (Figure 8). For example, the strength (i.e., Δarea) of *V*_ramp_-induced *I*_h_ in the control period (i.e., absence of CRS) was 4.16 ± 0.31 mV⋅nA (*n* = 8). However, after exposure to 10 or 30 μM CRS for 1 min, the value of Hys_(V)_’s Δarea in GH_3_ cells was reduced significantly to 3.24 ± 0.25 mV⋅nA (*n* = 8, *p* < 0.05) or 2.92 ± 0.22 mV⋅nA (*n* = 8, *p* < 0.05), respectively. OXAL, a chemotherapeutic drug, has been shown to activate *I*_h_ (Resta et al., 2018; Chang et al., 2020; Yongning et al., 2021). In cells exposed continuously to 30 μM CRS, the subsequent application of OXAL (10 μM) reversed the CRS-mediated decrease in the Δarea to 4.23 ± 0.28 mV⋅nA (*n* = 8, *p* < 0.05).

### 3.8. Predicted docking interaction of the CRS molecule with the HCN channel

**FIGURE 9:**
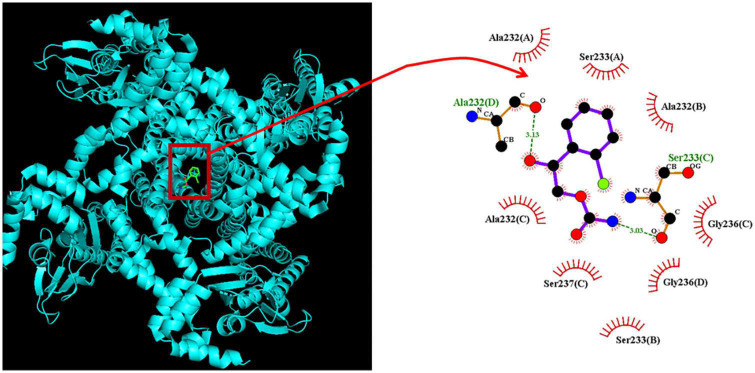
Predicted docking between the CNG (cyclic nucleotide-gated) channel and the carisbamate (CRS) molecule. **(Left panel)** Depicts the CNG channel protein structure acquired from RCSB PDB (PDB ID: 5V4S) and the CRS molecular structure acquired from PubChem (Compound CID: 6918474). PyRx software (https://pyrx.sourceforge.io/) (accessed on 28 April 2023) was used to show that the HCN channel structure can be properly docked by the CRS molecule. **(Right panel)** Depicts an expansion of the red box in the **left panel** arrow. This diagram of the interaction between the CNG channel and the CRS molecule was generated using LigPlot^+^ (https://www.ebi.ac.uk/thornton-srv/software/LIGPLOT/) (accessed on 28 April 2023). The red arcs over which spokes radiate toward the ligand (i.e., CRS) represent hydrophobic contacts, while the green dotted lines indicate hydrogen bond formation. The graph in the **right panel** indicates an expanded display of red box with a curve arrow in the left side.

We wanted to study how CRS could interact with the HCN channel protein, but since it is not known so far the isoform(s) blocked by the CRS, we have used the structure of a closely related CNG (cyclic nucleotide-gated) channel. Information from the provider of the original structure data from PDB (James et al., 2017) revealed several close relationships between the cryostructure of CNG and HCN. The CNG channel protein structure was obtained from PDB (PDB ID: 5V4S); we used PyRx software to see how the CRS molecule could dock into the channel protein. Figure 9 presents the predicted docking sites of CRS for interaction with amino acid residues of the CNG channel. Specifically, the CRS molecule was predicted to form hydrophobic contacts with the CNG channel residues Ala232(A), Ala232(B), Ala232(C), Ser233(A) Ser233(B), Gly236(C), Gly236(D), and Ser237(D), as well as hydrogen bonds with residues Ala232(D) and Ser232(C), with estimated distances of 3.15 and 3.05 Å, respectively. It is likely that the CRS molecule interacts with the transmembrane region (position 211–238) or membrane segment (position 210–235 or 211–231) of the CNG channel, although details of these binding sites remain unclear. Moreover, as shown in Supplementary Figure 1, the docking interaction of the CRS molecule with the hNa_V_1.7 channel was generated in the Supplementary information.

## 4. Discussion

The current study has yielded several main findings. First, CRS can exert the inhibitory effect on *I*_Na_ which can vary depending on the concentration applied, time course of the current, and stimulus frequency used. Second, exposure to CRS led to differential suppression of *I*_Na(T)_ and *I*_Na(L)_ activation in response to a brief step depolarization, with effective IC_50_ values of 56.4 and 11.4 μM, respectively. Third, CRS failed to alter the steady-state *I–V* relationship of *I*_Na(T)_ in GH_3_ cells but decreased the decay time constant of cumulative *I*_Na(T)_ inactivation during pulse train stimulation. Fourth, DLT-mediated enhancement of *I*_Na_ was attenuated by subsequent exposure to CRS. Fifth, CRS elicited a concentration-dependent decrease in *I*_h_ amplitude, with an IC_50_ value of 38 μM. Sixth, CRS suppressed the Hys_(V)_ strength of *I*_h_ activation in response to a long-lasting isosceles-triangular *V*_ramp_. Collectively, these results demonstrate that CRS-mediated changes in the magnitude, gating properties, and frequency dependence of *I*_Na_ and in the magnitude of *I*_h_ and Hys_(V)_ can modify the functional activities of electrically excitable cells.

The IC_50_ value required for CRS-mediated inhibition of *I*_Na(T)_ or *I*_Na(L)_ was lower than the concentration used to suppress the evoked action potentials in previous studies of piriform cortical neurons or hippocampal neurons (Liu Y. et al., 2009; Whalley et al., 2009). Unlike a previous report showing inability of CRS to alter *I*_Na(L)_ measured from rat hippocampal neurons (Liu Y. et al., 2009), the IC_50_ value required for CRS-mediated inhibition of *I*_Na(T)_ in rat hippocampal neurons was estimated to be around 100 μM (Liu Y. et al., 2009), which is greater than the IC_50_ values that we estimated for the suppression of *I*_Na(T)_ or *I*_Na(L)_. Therefore, during cell exposure to CRS at pharmacologically achievable concentrations, it is plausible to assume that CRS-induced inhibition of *I*_Na_ is an important ionic mechanism underlying the perturbing effects of the drug on membrane excitability.

In this study, the exponential decline of *I*_Na(T)_ during a 20-Hz train of depolarizing pulses (i.e., 40-ms depolarizing pulses from −80 to −10 mV at 20 Hz for 1 s) became pronounced with exposure to CRS. The results reflect the frequency dependence of *I*_Na(T)_ during pulse train stimulation, consistent with recent studies (Huang et al., 2015; Navarro et al., 2020; Wu et al., 2022a). As a result, exposure to CRS would lead to a loss of function due to changes in the time-dependent current inactivation. Therefore, the CRS-mediated decrease in *I*_Na(T)_ is closely linked to the strong frequency-dependent decrease in *I*_Na(T)_ during pulse train stimulation (Navarro et al., 2020).

An earlier report showed that CRS could suppress the magnitude of a T-type Ca^2+^ current (Kim et al., 2017). Therefore, we postulated that in GH_3_ cells, a CRS-induced block of *I*_Na_ could be due, in part, to the inhibitory effect of the drug on the voltage-gated Ca^2+^ currents that are functionally expressed in excitable cells (Wu et al., 2003; Stojilkovic et al., 2010). However, under our experimental conditions, the voltage-gated inward currents were either sensitive to stimulation by Tel and DLT or were suppressed by TTX. Tel and DLT were previously shown to activate *I*_Na_ (Chang and Wu, 2018; Lai et al., 2020; Bothe and Lampert, 2021; Lin et al., 2022), while TTX is a potent inhibitor of *I*_Na_. Under continuous exposure to CRS, exposure to DLT strikingly counteracted the CRS-induced decrease in the instantaneous *I*_Na(W)_ in GH_3_ cells, and exposure to Tef reversed the CRS-mediated reduction of the decaying time constant of *I*_Na(T)_ inactivation in response to PT depolarizing stimuli. Alternatively, neither CdCl_2_ nor nimodipine effectively inhibited the inward currents in GH_3_ cells. These results prompt us to reflect that, *I*_Na_ (*I*_Na(T)_, *I*_Na(L)_ and *I*_Na(W)_) is susceptible to blockade by CRS, and that this drug exerts stronger suppressive effects on *I*_Na(L)_ than on *I*_Na(T)_. The action of CRS on excitable membranes cannot be explained solely by its ability to suppress the magnitude of a T-type Ca^2+^ current.

CRS was shown to increase Cl^–^ conductance in piriform cortical neurons (Whalley et al., 2009). In our study, the *I*_h_ amplitude was not inhibited by chlorotoxin (1 μM) but was inhibited by ivabradine (10 μM) (data not shown). Similarly, chlorotoxin did not suppress the amplitude of *I*_Na(T)_ or *I*_Na(L)_ in tested cells. Chlorotoxin is known to suppress Cl^–^ channels. It is therefore tempting to speculate that CRS-induced inhibition of *I*_Na_ or *I*_h_ is not associated with an increase in Cl^–^ channel activity.

In this study, exposure to CRS led to a change in *I*_h_ in a concentration-dependent manner, with an IC_50_ value of 38 μM. There also was a marked retardation of the activation time course (i.e., an increase in the τ_act_ value) of *I*_h_ in response to a 2-s hyperpolarizing command voltage. Increasing evidence suggests that the strength of non-equilibrium Hys_(V)_ in *V*_ramp_-evoked *I*_h_ plays an essential role in modifying electrical behavior (e.g., action potential firing) in various types of excitable cells (Mãnnikkõ et al., 2005; Fürst and D’Alvanzo, 2015; Peters et al., 2021, 2022; Wu et al., 2022b). In our study, exposure to CRS also suppressed the Hys_(V)_ of *I*_h_ activation during the triangular *V*_ramp_, indicating that this drug is likely to interact with the voltage-sensing domains of the HCN channel.

There are four mammalian isoforms of HCN, namely HCN1, HCN2, HCN3, and HCN4, which constitute the macroscopic *I*_h_ (or *I*_f_) (Fürst and D’Alvanzo, 2015; Peters et al., 2022). HCN2, HCN3, and mixed HCN2+HCN3 channels are abundantly expressed in GH_3_ cells and other types of endocrine and neuroendocrine cells (Kretschmannova et al., 2006, 2012). We emphasize that *I*_h_ has been demonstrated to be functionally present in heart cells (Belardinelli et al., 1988; Irisawa et al., 1993; Depuydt et al., 2022). Therefore, the CRS-mediated inhibition of *I*_h_ seen in excitable cells is very likely to be responsible for the drug’s ability to attenuate an increase in heart rate induced by exposure to organophosphate administration, as described previously (Deshpande et al., 2016a,b; Deshpande and DeLorenzo, 2020). Apart from the ability of CRS to inhibit *I*_Na_, as detailed above, CRS-mediated changes in the magnitude, gating kinetics, and Hys_(V)_ behavior of *I*_h_ also may be of pharmacological or therapeutic relevance.

In dendritic synapses, *I*_h_ has a shunt mechanism that paces the excitatory post-synaptic potential amplitude and duration and limits temporal summation (Magee, 1998, 1999). HCN2 is strongly expressed in the thalamus, where it is involved in the spontaneous firing of thalamocortical neurons in oscillation or in the 3-Hz spike and wave pattern (Poolos, 2004). A study of human subjects revealed a role for HCN2 in susceptibility to juvenile myoclonic epilepsy (Wu et al., 2018). HCN2-knockout animals have been reported to develop spontaneous absence seizures and cardiac sinus arrhythmia (Poolos, 2004; Heuermann et al., 2016). Dendritic HCN1 and HCN2 channels were found to be downregulated in a pilocarpine model of epilepsy, while in the chronic period, expression of these channels increased (Jung et al., 2007). The differential regulation of *I*_h_ in different seizure models suggests that a region-specific density of *I*_h_ may serve various purposes in epileptogenesis and may play a role in protection against or resistance to seizures. Furthermore, an increased *I*_h_ may co-exist with, and possibly contribute to, persistent dendritic hyperexcitability following febrile seizures in the hippocampus, in contrast to the exclusively anti-convulsive role often attributed to an increased *I*_h_ (Dyhrfjeld-Johnsen et al., 2008). Interestingly, the inhibitory action of *I*_h_ may be caused by its interaction with the delayed-rectifier M-type K^+^ current, which can enhance or inhibit spike firing in response to an excitatory post-synaptic potential when the spike threshold is low or high, respectively (George et al., 2009). Additionally, the coupling of *I*_h_ to A-type K^+^ channels was shown to regulate neuronal excitability (Mishra and Narayanan, 2015). Further studies are needed to clarify the concerted effects of CRS on *I*_h_ and *I*_Na_ in epileptogenesis.

Finally, the modulatory actions of CRS on ion channels demonstrated in this report are not limited to the interactions of this drug with Na_V_ channels. The inhibitory effects of CRS on *I*_Na(T)_, *I*_Na(L)_, *I*_Na(W),_ and *I*_h_ are also expected to constitute important underlying mechanisms through which the drug exerts concerted effects on cellular function and excitability.

## Data availability statement

The original contributions presented in this study are included in the article/Supplementary material, further inquiries can be directed to the corresponding authors.

## Author contributions

T-YH, S-NW, and C-WH designed the experiment, analyzed the data, and wrote the manuscript. S-NW and C-WH performed the experiments and created the figures. All authors contributed to the article and approved the submitted version.
