# Supplementary material for: Concerted suppressive effects of carisbamate, an anti-epileptic alkyl-carbamate drug, on voltage-gated Na+ and hyperpolarization-activated cation currents
Source: Front Cell Neurosci. 2023 May 24;17:1159067. doi: 10.3389/fncel.2023.1159067 (PMC10244622; doi:10.3389/fncel.2023.1159067)
Supplement: Supplementary file 1 [file Data_Sheet_1.docx]

**Supplementary Information**

*Molecular docking on the interaction between hNa_V_1.7 channel and carisbamate (CRS)*

In this study, we also investigated how the protein of the hNa_V_1.7 channel was optimally docked with the CRS molecule by using PyRx software. The protein structure of hNa_V_1.7 was taken from RCSB (PDB ID: 5EK0). The predicted docking sites of the CRS molecule, with which the amino-acid residues can interact, are presented in Supplementary Figure 1. The CRS molecule was found to form hydrophobic contacts with certain amino acids, such as Thr1678(C), Thr1678(D), Leu1679(A), Leu1679(D), Glu1680(A), Glu1680(C), and Glu1680(D). The atoms in the CRS molecule also have several hydrogen bonds with residue Leu1679(C) at 2.80 A, Ser1681(D) at 2.97 and 3.04 A, Glu1680(C) at 2.97 A, and Ser1681(A) at 2.97 and 3.03 A. The CRS molecule may dock the transmembrane region (position: 1597-1613) of hNa_V_1.7 channel (PDB: 5EK0) with a binding affinitiy of -6.9 kcal/mol. The results thus reflected the possibility that, apart from its ability to bind to HCN channel (indicated in **Figure 9**), the CRS molecule could potentially dock to the hNa_V_1.7 channel.


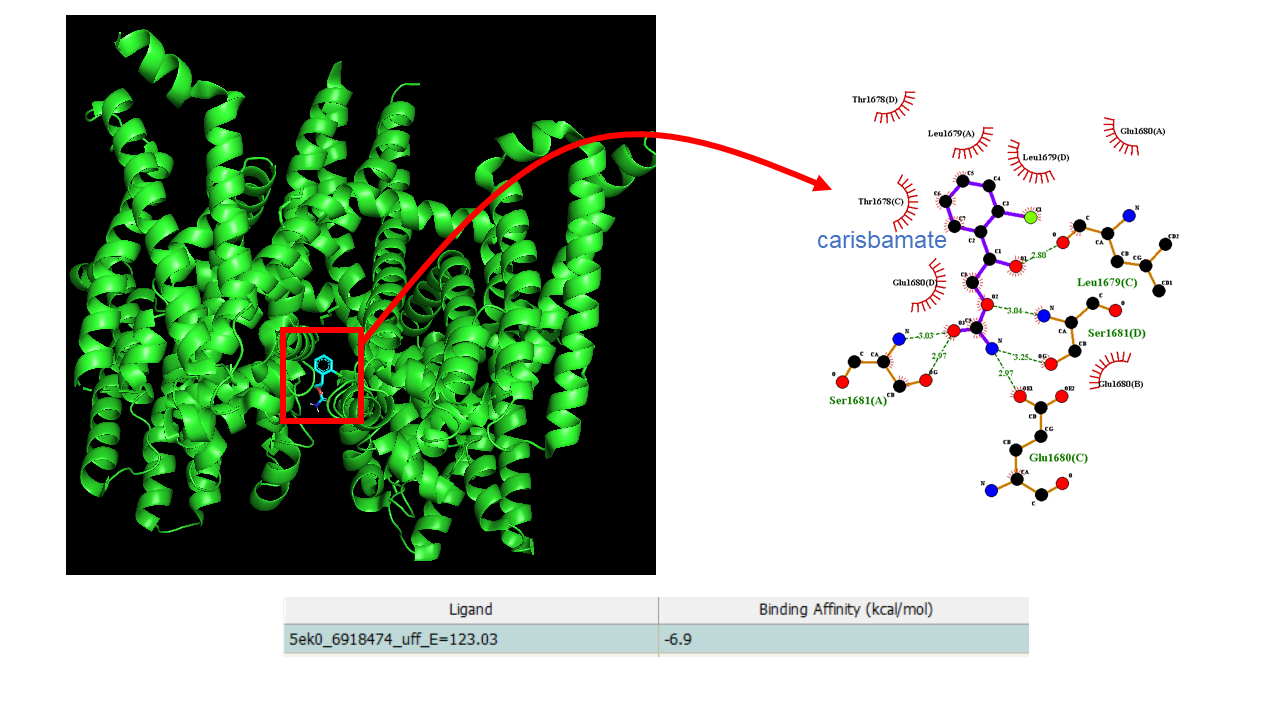


**Supplementary Figure 1.** Results on molecular docking between the hNa_V_1.7 channel and the carisbamate (CRS) molecule. The protein structure of hNa_V_1.7 was obtained from RCSB (Research Collaboratory for Structural Bioinformatics) PDB ID: 5EK0, whereas the CRS molecule was from PubChem (Compound CID: 6918472 [3D conformer]). The structure about the hNa_V_1.7 channel was docked by the CRS molecule with PyRx software (indicated in left part). The interaction between the hNa_V_1.7 channel and the CRS molecule was created from the LigPlot^+^ (indicated in right side). Of note, the red arcs on which small bars radiated toward the ligand (i.e., CRS) represent hydrophobic interactions, while the green dotted lines residing in amino-acid residue (i.e., Leu1679(C), Glu1680(C), Ser1681(A), Ser1681(D)) show formation of hydrogen bonds.
